# The Influence of Co Addition on the Structure and Mechanical Properties of Tungsten Heavy Alloys

**DOI:** 10.3390/ma15249064

**Published:** 2022-12-19

**Authors:** Paweł Skoczylas, Mieczysław Kaczorowski

**Affiliations:** Department of Mechanics and Armament Technology, Faculty of Mechanical and Industrial Engineering, Warsaw University of Technology, Narbutta 85, 02-524 Warsaw, Poland

**Keywords:** tungsten heavy alloys, cobalt addition, mechanical properties, microstructure, powder metallurgy

## Abstract

This study shows the results of Ni replacement with Co in a W-Ni-Co-type tungsten heavy alloy (WHA) in terms of the structure and mechanical properties. Five alloys containing 92 wt. % of tungsten plus Ni and Co changing in the proportions (Co:Ni) of 1:9, 2:8, 3:7, 4:6, and 5:5 were prepared using liquid phase sintering (LPS). The specimens were studied directly after sintering and after solution heat treatment. The tensile strength, yield strength, and elongation were evaluated. The results of tensile tests were supplemented with microhardness measurements of tungsten grains and matrix. Light microscopy was used for the microstructure, and a scanning electron microscope (SEM) equipped with an EDS attachment was applied for the assessment of the fracture mode and chemical microanalysis. It was concluded that the replacement of Ni with Co led to a tensile property increase that was accompanied by a gradual decrease in elongation that started to be critical for a Co:Ni ratio higher than 4:6.

## 1. Introduction

For centuries, the continual competition between projectiles and armour occurred. From the beginning, there was the race between the spear (bolt) and target, as well as more recently between projectiles and armour. In the latter case, the competition especially concerns the tanks that are equipped with increasingly more advanced armour plates with special geometry antitank projectiles. Currently, the most common antitank projectiles are fin stabilised armour piercing discarding sabot (FSAPDS)-type projectiles, often called kinetic energy penetrators (KEP) [1,2,3,4,5,6,7,8]. The name reflects the mode of interaction of the projectile with the target that is destroyed because of extremely high kinetic energy accumulated in projectiles. This energy follows from the as-great-as-possible weight of the projectile core, which is made from a high density of material and extremely high velocity of the projectile, reaching 1600 m/s. The measurement of effectiveness of the projectile is depth of armour penetration, which is a function of material and velocity. The development of KEP is concentrated on the geometry and material used for it. In the case of projectile geometry, a simultaneous increase in the length to diameter ratio is observed. Until recently, depleted uranium (DU) was used as a material for penetrators, but because of its radioactivity, it has been replaced with tungsten heavy alloys (WHA) [8,9,10,11]. Many tungsten alloys have been developed, but among them, W-Ni-Fe-type alloys are the most popular [12,13,14]. However, very high demands concerning depth of armour penetration push researchers to look forward for alloys with extremely mechanical properties. This means that not only a high strength and hardness of WHA is needed but also a high value of fracture toughness must be assured [15,16,17,18]. The mechanical properties of WHA are a function of the microstructure parameters, including tungsten grain size, grain shape, proportion of tungsten grains to the matrix, the size of the direct contact of tungsten grains (the so-called “contiguity”), and the chemical composition of the matrix [19,20,21,22,23,24,25,26,27,28]. An increase in the percentage of tungsten in WHA causes a decrease in the proportion of matrix to tungsten grains and an increase in the theoretical density. An increase in the amount of tungsten in the alloy causes an increase in hardness, but after exceeding its optimal amount, the strength, plasticity, and impact properties decrease. Small-sized tungsten grains increase the strength and ductility of WHA. Larger tungsten grains increase the impact toughness at the expense of lowering strength. The shape of the grains is essentially spheroidal, but after exceeding the 90% tungsten content in the alloy, it is difficult to maintain such a shape without creating voids in the material, and therefore the grains begin to assume an irregular shape during the sintering process. The contiguity (CTG) parameter determines the share of the direct contact area of tungsten grains in relation to the total area of all grain boundaries and interfaces. Contiguity is a function of not only the tungsten content but also the temperature and time of the sintering process, as well as the heat treatment after sintering. An increase in the value of “contiguity” causes an increase in the number of direct contact points of tungsten grains with low bond strength, which reduces the strength, ductility, and impact toughness of the alloy. Post-sintering heat treatment is carried out to improve the strength, ductility, and impact strength of WHA [28,29,30,31,32,33,34]. The treatment is carried out in the temperature range of 1000–1200 °C for 1 to 24 h in nitrogen, argon, or vacuum atmosphere combined with cooling in water or oil. We improve the properties by, among others, removal of hydrogen dissolved in the matrix, as well as dissolution of impurities and intermetallic phases and carbides. Cobalt can positively affect the improvement of microstructure parameters [35,36,37,38,39,40,41,42,43,44,45,46,47], and thus we carried out this study on W-Ni-Co-type heavy alloys that appeared promising as a penetrator in FSAPDS ammunition.

## 2. Experimental Procedure

**Figure 1:**
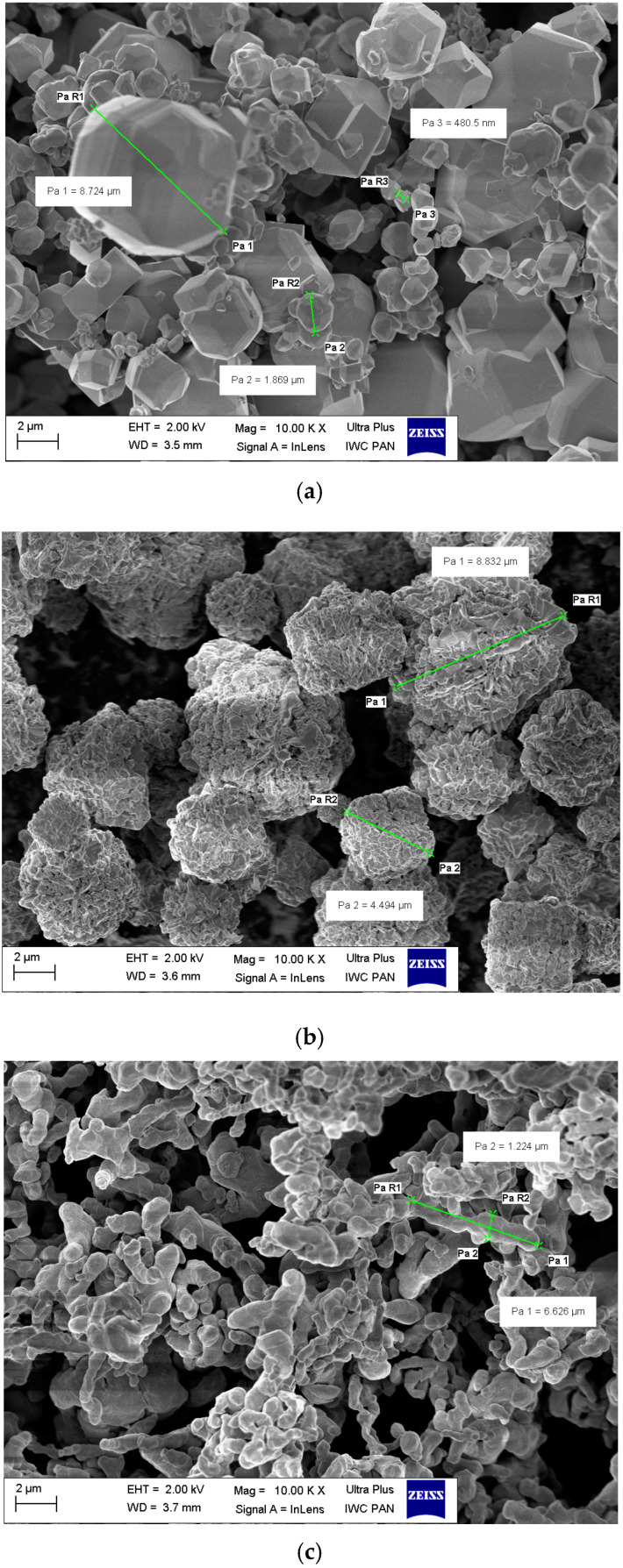
The morphology of powders: (**a**) tungsten, (**b**) nickel, and (**c**) cobalt (SEM).

To assure high density of the alloy, a tungsten heavy W-Ni-Co alloy with a 92 wt. % of tungsten was chosen. The concentration of nickel and cobalt as alloying elements were changed. The chemical composition of the five tungsten heavy alloys being studied is provided in Table 1. Being near the same density of Ni and Co assures a constant density of WHA. The high purity powders that are characteristic are shown in Table 2, and the morphology depicted in Figure 1 was used for preparing of the alloys. The powder was mixed for 20 h in a drum agitator and then compacted in steel die under pressure of 200 MPa. The weight of the bar-shaped green compact was 1800 g, and the length was 600 mm. The compacts were liquid phase sintered for 20 min at 1530 °C in a hydrogen atmosphere [30,31,32,33,34] in a Vacum Industries chamber furnace (Vacuum Industries, Inc., Somerville, MA, USA).

Finally, the compacts in the shape of rods of 510 mm in length and 15.2 mm diameter were heat treated for 3 h at 1200 °C in a vacuum furnace (CZYLOK Company, Jastrzębie-Zdrój, Poland) and then the water was cooled. The shrinkage of the sintered compacts, calculated on the basis of the change in the linear length of the bar, was approximately 18%. This heat treatment was carried out for hydrogen removal. The density measurements were carried out with the Archimedes method. The tensile test experiments were performed on quintuple specimens of 3 mm diameter according to the PN-EN 10002-1 standard to evaluate tensile strength, yield strength, and elongation. The tensile test was carried out on an INSTRON model 1115 testing machine (Instron, Norwood, MA, USA) with a strain rate of 7.2 × 10^−4^ s^−1^. To determine the Young’s modulus, we used an extensometer installed on a strength sample. Hardness of alloys was evaluated on the specimen in a parallel plane and perpendicular to the rod axis using the HRC method according to the PN-91/H-04355 standard on an HRC hardness tester machine (HR-150A, Jinan Hensgrand Instrument Co., LTD., Jinan, China). The hardness measurements were supplemented with microhardness testing that was performed with a Future-Tech FM 810 (Future-Tech Corp., Tokyo, Japan) tester under load and time of F = 0.245 N and 15 s, respectively. Structure observations included light metallography and scanning electron microscopy investigations were carried out on a Nicon-Eclipse MA 200 Microscope (Nikon Corporation, Tokyo, Japan) and on a microscope (Carl Zeiss Microscopy GmbH, Jena, Germany) by Zeiss Ultra Plus. Moreover, the chemical microanalysis was conducted with the EDS method on SEM microscopy with an attached EDS system (Quantax 400) by Bruker (Billerica, MA, USA). XRD measurements were performed on a Siemens D500 X-ray diffractometer in Bragg–Brentano (BB) geometry with CuKa radiation at the wavelength of λ = 0.15418 nm. The XRD studies were carried out with the goniometer speed of 0.5 °/min. The evaluation of Ni-based lattice constant was based on a {222} reflex position.

## 3. Results

### 3.1. Results of the Density Measurements

The results of the density measurements are provided in Table 3. As is seen from Table 3, all alloys possessed almost the same density, although they were a little smaller than the theoretical calculation.

### 3.2. Microstructure Observations

The structure study included metallographic investigations: both a conventional metallographic observation and a qualitative assessment. For the qualitative evaluation of microstructure elements, the NIS elements imaging software by Nicon was used. For both tests, specimens with a metallographic section parallel (R) and perpendicular (P) to the main axis of the bar were used. The quantitative analysis of the WHA microstructure was to determine the size of the tungsten grains, the percentage of the matrix, the elongation of the grains, and the share of the direct contact points of the tungsten grains—contiguity.

#### 3.2.1. Results of the Metallographic Observation

**Figure 2:**
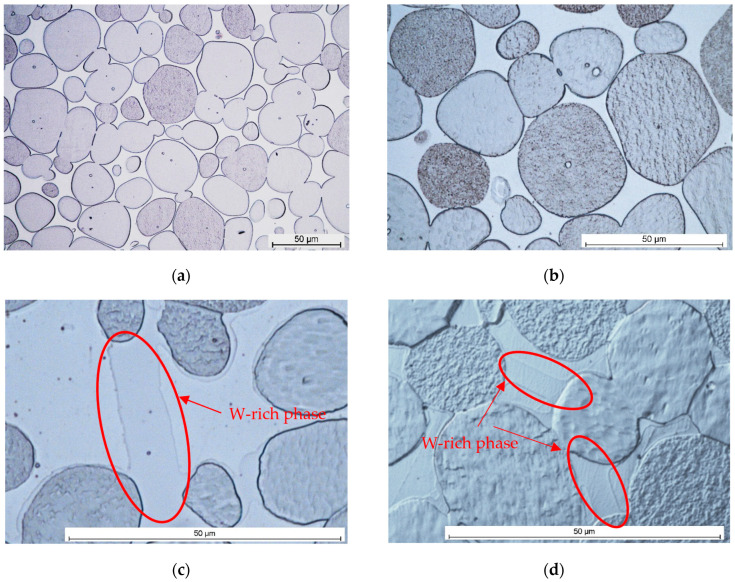
Microstructure of WHA samples: (**a**,**b**) WNi9Co1; (**c**,**d**) WNi5Co5.

**Figure 3:**
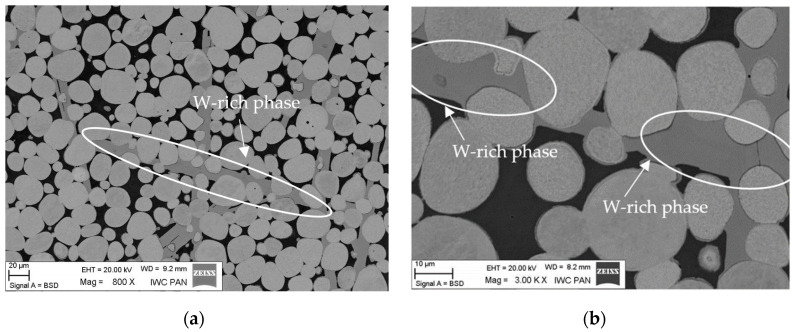

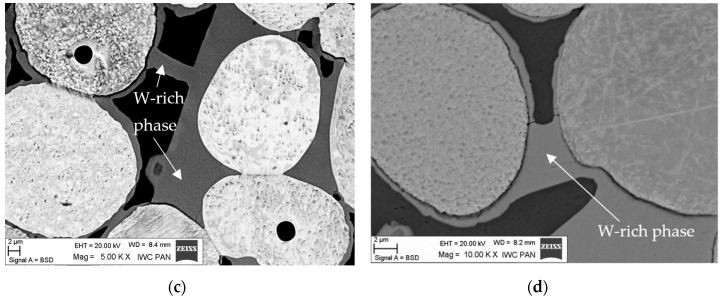
SEM images of the microstructure of the WNi5Co5 sample. Arrows and white circles indicate the position of the W-rich phase. Magnification: (**a**) 800×, (**b**) 3000×, (**c**) 5000×, (**d**) 10,000×.

The typical microstructure of the WNi9Co1 and WNi5Co5 alloys were observed in a conventional light microscope (Figure 2) and a scanning electron microscope (Figure 3). In both figures, spheroidal tungsten grains in an Ni-based matrix were visible. Apart from them, a W-rich phase appeared, which is marked with arrows (Figure 2). The same phase was grey in photos taken in SEM (Figure 3) where backscattered electrons (BSEs) were used for imaging.

It is worth noting that this phase occurred in the shape of needle-shaped precipitates and formed a shell on the tungsten grains (Figure 3d).

#### 3.2.2. Results of the Qualitative Assessment of the Microstructure

The results of the qualitative measurements are included in Table 4. The number of tungsten grains analysed in a single measurement was 250 on average.

The presented results did not indicate any significant dependencies between the considered metallographic section for a single bar and the Co:Ni ratio for all bars. It appears from Table 4 that participation of tungsten grains in a structure of WNi5Co5 appeared to be somewhat smaller, and the contiguity in WNi9Co1 was also smaller than other WHAs.

#### 3.2.3. Fractographic Investigations

**Figure 4:**
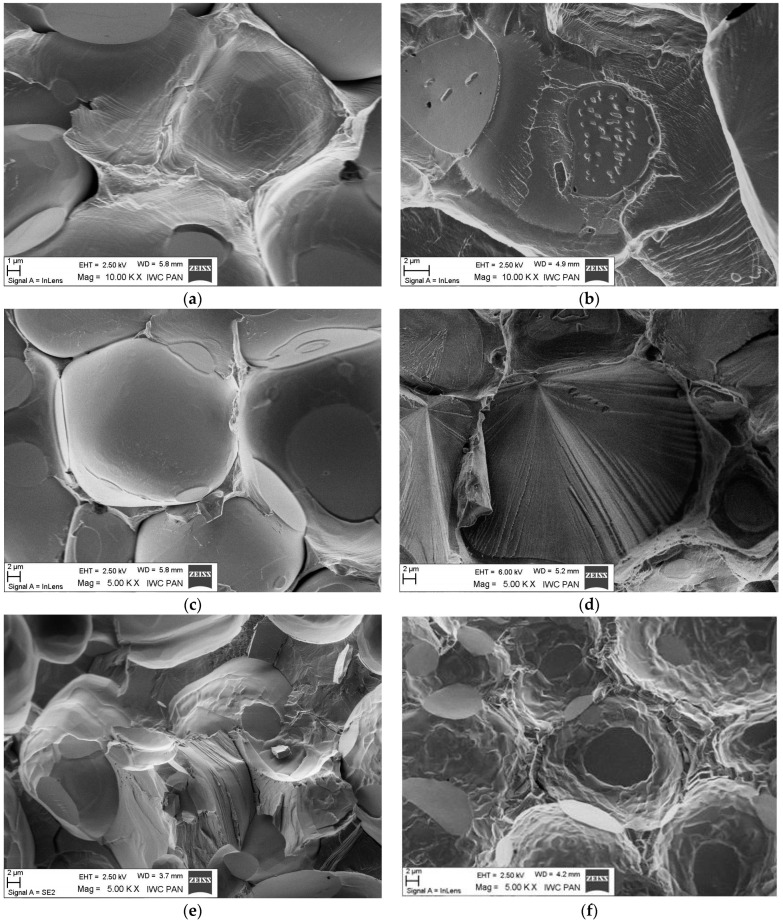
SEM images of the fracture surface of the alloys: WNi9Co1, WNi7Co3, and WNi5Co5 directly after sintering (**a**,**c**,**e**) and after heat treatment (**b**,**d**,**f**). Magnification: (**a**,**b**) 10,000×, and (**c**–**f**) 5000×.

The aim of the fractographic investigation was to evaluate the mode (character) of failure. Figure 4 illustrates the examples of the fracture surfaces of the alloys WNi9Co1, WNi7Co3, and WNi5Co5 obtained after the tensile test. The images in the left column show the nature of the fractures in the WHA samples immediately after sintering, while the images in the right column show examples of fracture morphology of the samples after heat treatment.

As can be seen, the fracture surface immediately after sintering was intergranular and brittle; there was no sign of plasticity (Figure 4a,c,e). In the case of the WNi5Co5 alloy, small precipitates were detected on the fracture surface (Figure 4e). The nature of the cracking of samples changed significantly after heat treatment. In WNi9Co1 and WNi7Co3 alloys, a brittle cleavage plane in the tungsten grain was identified (Figure 4b,c). Moreover, in the case of the first one (Figure 4b), small precipitates appeared on the flat surfaces, which were tungsten grain contact points. No such precipitates were visible in the fracture surface of the WNi5Co5 alloy. In this alloy, an almost continuous shell on tungsten grains was identified (Figure 4f).

### 3.3. Chemical Analysis

**Figure 5:**
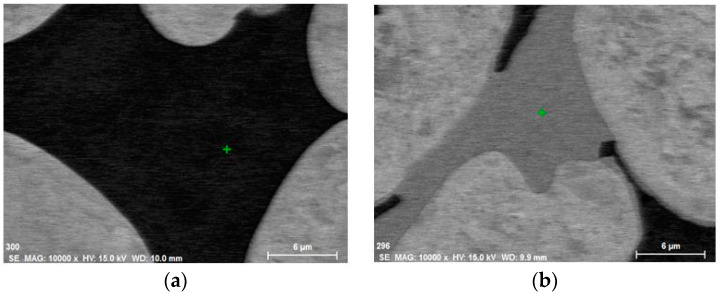
Illustration of places where EDS analyses were carried out: (**a**) matrix; (**b**) W-rich phase. The point where the chemical composition was analysed is marked with a green cross.

The goal of chemical analysis was to verify if and how much Co addition influenced the chemistry of WHA, particularly in terms of the potential solubility of cobalt in a Ni-based matrix. The places of EDS analysis are shown in Figure 5, and the results of investigations are included in Table 5.

**Figure 6:**
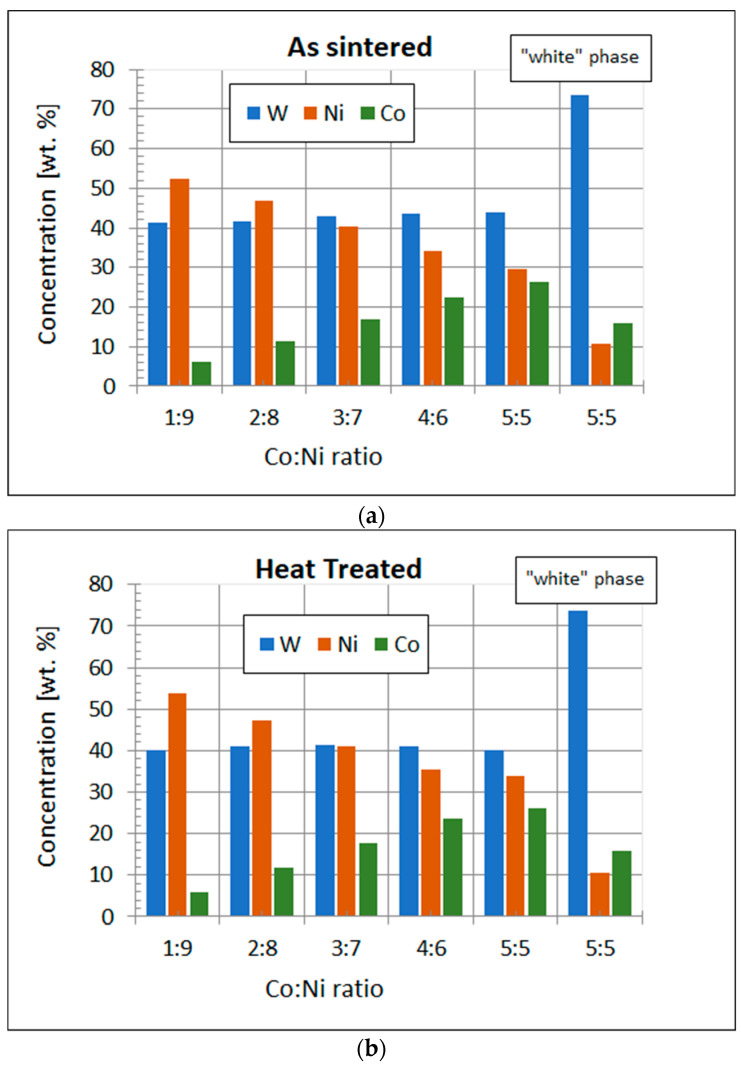
The concentration of W, Ni, and Co in alloys with different Co:Ni ratios: (**a**) as sintered; (**b**) heat treated for 3 h at 1200 °C.

Figure 6 shows the concentration of W, Ni, and Co in the matrix of the alloys with different Co to Ni ratios. It should be noted that the results concerning the WNi5Co5 alloy on the graph (Figure 6) are provided for the matrix and W-rich phase. In all types of alloys, tungsten concentration in the matrix was almost on the same level, while Ni content decreased simultaneously with an increase in Co concentration. There was only a substantial difference between tungsten concentration in the matrix and in the W-rich phase. The average tungsten content in the matrix of all alloys was at the level of 40 wt. %. The average concentration of tungsten of the W-rich phase exceeded 70 wt. %.

### 3.4. Results of the X-ray Diffractometry Analysis

**Figure 7:**
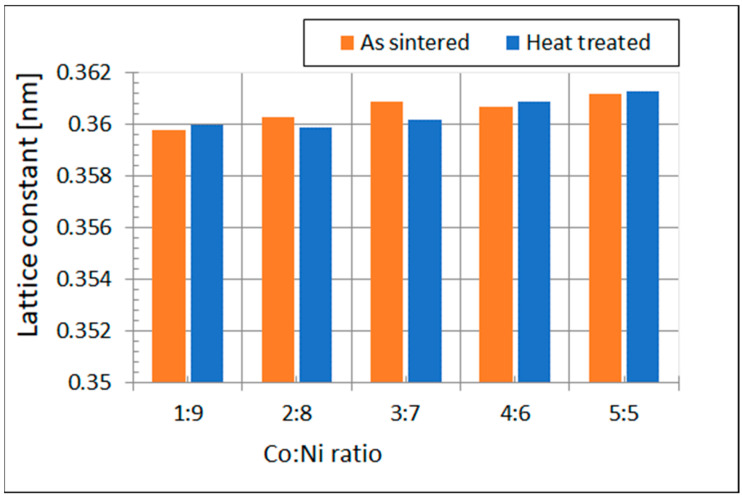
The changes of the Ni base matrix lattice constant.

The X-ray diffractometry studies were carried out to observe the eventual changes of the lattice constant of the Ni base matrix caused as result of Co solubility in the matrix. The results of the lattice constant calculation based on the X-ray study are provided in Table 6, and the graph illustrating the changes of the lattice parameter of the matrix are depicted in Figure 7. The estimated error of the lattice constant measurement did not exceed ±0.0002 nm.

As lattice constant of pure Ni was equal to 0.3499 nm, it follows from Table 6 and Figure 7 that the introduction of cobalt caused a small increase in the lattice parameter. It is also easy to distinguish some disruption in the course of the lattice constant increase. It can be suspected as a result of the W-rich phase formation.

### 3.5. Results of Mechanical Testing

The results of the tensile test were carried out to evaluate tensile stress—R_m_, yield stress—R_p 0.2_, and elongation—A_5_. The results of the investigation are provided in Table 7, where HRC and Young’s modulus data are included. The results were obtained on a specimen after sintering and heat treatment, including 3 h annealing at a temperature of 1200 °C in a vacuum furnace and water quenching. The research was carried out on three samples from each state (Table 7).

**Figure 8:**
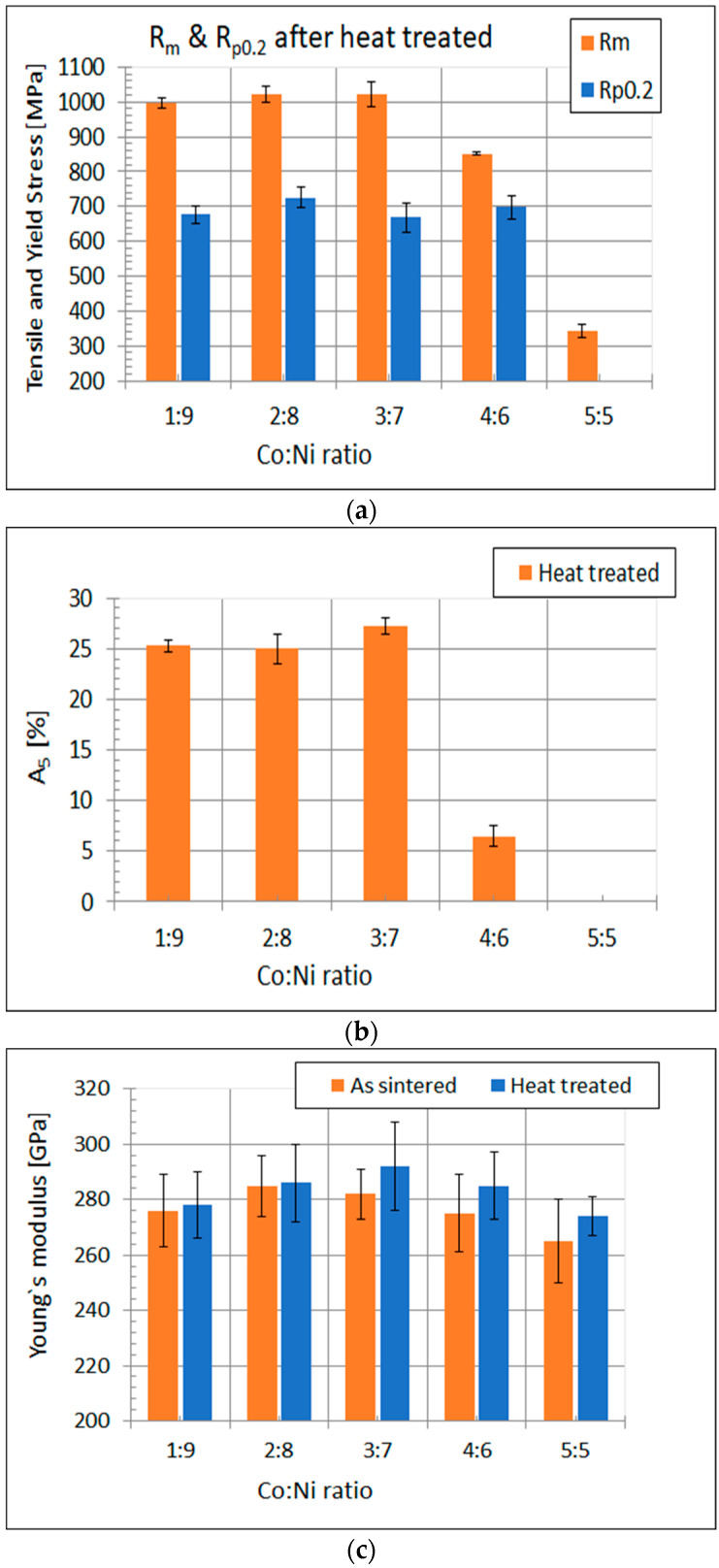
The changes of properties: (**a**) tensile strength and yield strength; (**b**) elongation; (**c**) Young’s modulus with a Co to Ni ratio.

The graphs illustrating the changes of mechanical properties for different alloys are presented in Figure 8.

It is clearly visible from Figure 8 that the increase in the Co:Ni ratio above 0.5 caused a substantial decrease in both tensile and yield strength (Figure 8a). For the Co:Ni ratio in the range of 1:9 to 3:7, no adverse effect of cobalt on the reduction of elongation was observed. For the ratio of Co:Ni equal to 4:6, a drastic reduction in elongation was observed. A further increase in the Co:Ni ratio caused the alloy elongation to drop to zero (Figure 8b).

**Figure 9:**
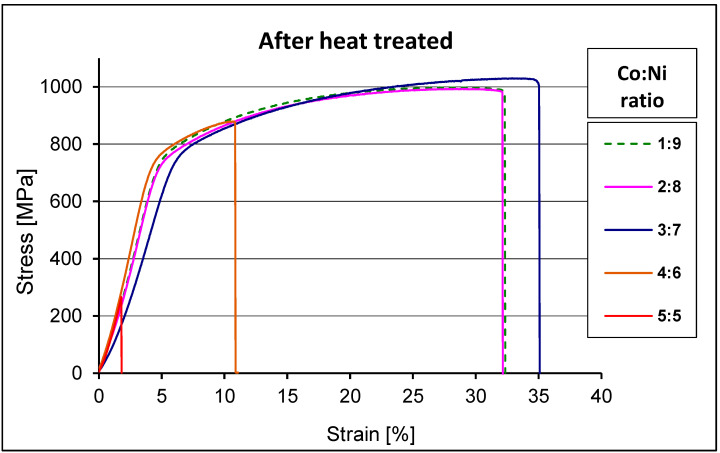
The stress–strain curves for all samples after heat treatments.

Figure 9 shows a typical example of stress–strain curves obtained for heat-treated alloys with different cobalt concentrations. The course of the graphs were very similar, except for the Co to Ni ratio equal to 5:5. The elongation of the alloy with the ratio Co:Ni equal to 4:6 was three times less than the other alloys. The difference in Young’s modulus of the alloys increased with an increase in the Co:Ni ratio.

### 3.6. Microhardness Testing

**Figure 10:**
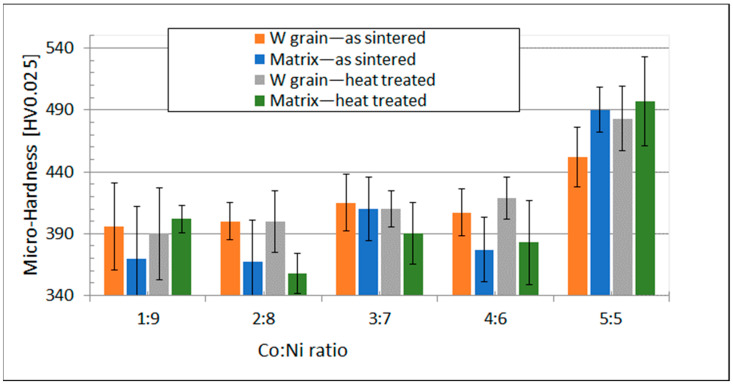
Change of hardness HV0.025 as function of the Co to Ni ratio.

Microhardness testing was carried out for tungsten grains and the Ni base matrix. At least 12 measurements were performed for each sample. The load F = 0.245 N and time 15 s were selected to assure the best results of the measurements. The results of microhardness testing are provided in Table 8, and its graphic illustration is found in Figure 10.

The graphic illustration of the hardness measurement is depicted in Figure 10.

It can be seen from the graph in Figure 10 that the microhardness of tungsten grains was somewhat higher than the Ni base matrix except for the Co to Ni ratio equal to 5:5. Although it was surprising, this could have been caused by the W-rich phase detected in the matrix whose microhardness reached over 1000 HV0.025, and it probably could increase matrix hardness (Table 8). It is worth noting the extremely high hardness of the W-rich phase that was double the tungsten grains (Table 8).

**Figure 11:**
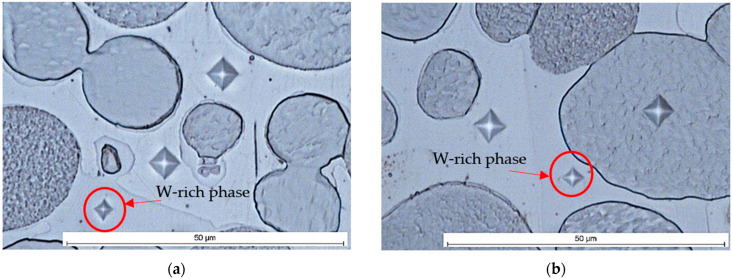
Comparison of indenter impressions in the sample WNi5Co5: (**a**) in the matrix and W-rich phase, (**b**) in the matrix, W-rich phase and tungsten grain (the indenter impression in the W-rich phase is marked with a red circle).

Figure 11 shows the microstructure of the tungsten alloy with a Co:Ni ratio equal to 5:5. It is easy to see the differences between the size of the indent in the tungsten grain or matrix and in the W-rich phase.

## 4. Discussion

Five WNiCo-type weight heavy alloys containing 92 wt. % tungsten plus the addition of nickel and cobalt with different proportions of Co to Ni equal to 1:9, 2:8, 3:7, 4:6, and 5:5 were studied. The aim of the study was to investigate how the addition of Co influences the structure and mechanical properties of WHA. The alloys were prepared from high-purity powders. The alloys were made by liquid phase sintered (LPS) in a hydrogen atmosphere and then were heat vacuum treated for 3 h at a temperature of 1200 °C. The heat treatment was carried out to remove hydrogen, causing hydrogen brittleness. The specimens cut from each alloy were subjected to tensile tests in order to evaluate tensile and yield strength and elongation either after sintering or after heat treatment. Moreover, the hardness of alloys and the microhardness of HV0.025 were investigated. The mechanical testing was supplied with the structure observations using conventional light and scanning microscopy (SEM). The SEM studies made fractography observations and chemical microanalysis by EDS attachment possible. To evaluate the changes of lattice constant with the addition of Co, X-ray diffractometry was used.

**Figure 12:**
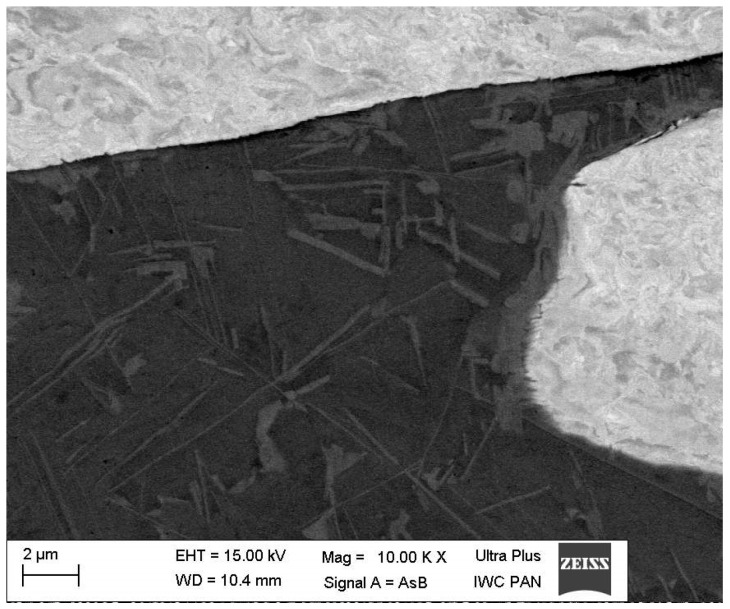
Fine needle-like precipitates in the matrix of the WNi6Co4 alloy (SEM); mag.—10,000×.

The density of alloys was almost the same as the theoretical one, which proved the lack of porosity. The results of the tensile experiment showed that the mechanical properties of the heat-treated alloys were much better than that of sintered alloys. The much lower tensile and yield strength and total lack of elongation of sintered alloys (Figure 8) compared with sintered and heat treated alloys resulted from hydrogen embrittlement. A rapid decrease in mechanical properties, particularly in terms of the elongation in heat-treated alloys with a Co:Ni ratio exceeding 0.5, was caused by the W-rich phase observed in the microstructure (Figure 2 and Figure 3). The W-rich phase started to appear in the matrix, probably in the WNi6Co4 alloy, and continued into the WNi5Co5 alloy, first as small needle-like precipitates (Figure 12) and then forming a continuous shell on a tungsten grain (Figure 3).

The microhardness of this phase was more than double that of tungsten grains or the matrix (Table 8). From the chemical analysis, the concentration of elements in atomic percent in the W-rich phase can be described as the Ni_2_Co_3_W_5_ phase that corresponded to the Co_7_W_6_ very brittle intermetallic phase in the W-Co equilibrium phase diagram [48,49,50]. It is worth noting that a similar hard phase with 75 wt. % of tungsten was discovered by Sengupta and coworkers who studied 90W-6Ni-4Co WHA modified with NiB [51]. If so, it can be proposed that this hard and brittle phase will break during loading, creating sharp edges working as stress concentrators. This destructive mechanism applies mainly to the needle-like precipitates and explains the dramatic influence of Co on mechanical properties of WHA with a higher Co:Ni ratio. As follows from SEM observations, the tungsten grains in the WNi5Co5 alloy were covered with an intermetallic W-rich phase (Figure 3) that remained on them after failure of the sample during the tensile test (Figure 4c). Such behaviour suggests that the strength between the intermetallic phase and tungsten grains was greater than with the matrix. Further, it can be suggested that the shell strengthened the tungsten grain by preventing transcrystalline cracking by cleavage.

It is worth noting to see the mechanism of structure evolution. In the case of relatively small cobalt addition, Co atoms replace Ni atoms in a solid solution, causing a small increase in Ni lattice constant (Table 6). A higher content of Co in an alloy exceeding solid solubility Co in a Ni-based matrix led to nucleation and growth of a very hard and brittle intermetallic W-rich phase. It initially formed fine needle-like precipitates that eventually grew into large rod-like particles or dendrites (Figure 3) and formed a shell on the tungsten grains. This caused a substantial decrease in strength properties and a dramatic decrease in elongation.

## 5. Conclusions

The results of the investigation presented above and the discussion make it possible to propose the following:

1. The parameters of the liquid phase sintering process allow for the obtaining of a homogeneous, non-porous material with an actual density comparable to the theoretical density.
2. The applied parameters of heat treatment consisting of annealing and cooling in water make it possible to change the properties of the WHA material from brittle to elastic–plastic.
3. Heat treatment increases the strength, plastic properties, hardness, and Young’s modulus.
4. Tungsten heavy alloys of Co to Ni with proportions of 3:7, 2:8, and 1:9 have similar mechanical properties.
5. An increase in Co concentration causes brittleness of alloys, which is especially visible in the case of WNi5Co5.
6. The brittleness is caused by the formation of the Ni_2_Co_3_W intermetallic phase, which is probably very close to the Co_7_W_6_ phase existing in the Co-W binary equilibrium diagram (μ phase). It can be suspected that the Ni_2_Co_3_W phase is really the (Co,Ni)_7_W_6_ intermetallic phase. This phase appears as a needle in the matrix and forms a thin shell on tungsten grains.
7. This intermetallic W-rich phase is very hard, which was found by microhardness measurements of the matrix that showed that its hardness was equal to 1000 HV0.025 and was double of that where the intermetallic phase did not exist.
8. The deviation from the equiaxials of tungsten grains is related to their accommodation during sintering in the process of contact flattening.
9. Increasing the proportion of cobalt to nickel increases the concentration of tungsten in the matrix.
10. Increasing the proportion of cobalt to nickel increases the parameter of the lattice constant of the matrix.
11. Heat treatment lowers the tungsten concentration in the matrix, which results in a reduction of the lattice constant parameter of the matrix.
12. Among the five alloys studied, two of them (WNi6Co4 and WNi5Co5) are too brittle to be promising for military applications, where extremally high fracture toughness is needed.
13. Three alloys, namely, WNi9Co1, WNi8Co2, and WNi7Co3, are planned for further testing involving heat treating and cold working.

## Figures and Tables

**Table 1:** Chemical composition of the alloys.

Alloy Designation	Chemical Composition (wt. %)						Co:Ni Ratio
	W		Ni		Co		
	wt. %	at. %	wt. %	at. %	wt. %	at.%	
WNi9Co1	92	78.6	7.2	19.3	0.8	2.1	1:9 = 0.111
WNi8Co2			6.4	17.1	1.6	4.3	2:8 = 0.250
WNi7Co3			5.6	15.0	2.4	6.4	3:7 = 0.429
WNi6Co4			4.8	12.8	3.2	8.6	4:6 = 0.667
WNi5Co5			4.0	10.7	4.0	10.7	5:5 = 1.000

**Table 2:** The characteristics of powders used for the mixture preparation.

Value Analysed	Powder		
	Tungsten	Nickel	Cobalt
Grain size (FSSS) (µm)	3.2	4.65	1.35
Bulk density (mg/m^3^)	5.34	2.22	1.43
Specific area BET (m^2^/g)	0.26	0.36	0.79
Particle size distribution (µm)			
D (0.1)	3.14	4.58	3.48
D (0.5)	9.53	11.89	8.04
D (0.9)	28.4	31.88	16.37

**Table 3:** Density of alloys.

Alloy Designation	Co:Ni Ratio	Density (mg/m^3^)	
		Theoretical (Calculated)	Measured
WNi9Co1	1:9	17.65	17.63
WNi8Co2	2:8		17.62
WNi7Co3	3:7		17.60
WNi6Co4	4:6		17.62
WNi5Co5	5:5		17.63

**Table 4:** Results of the WHA qualitative assessment carried out on specimens with metallographic parallel (R) and perpendicular (P) sections with respect to the main axis of the bar.

Co:Ni Ratio	Participation of Tungsten Grains in the Structure		Equivalent Diameter of Tungsten Grains		Elongation of Tungsten Grains		Contiguity	
	(%)		d_2_ (µm)		d_max_/d_min_			
	Surface							
	P ^1^	R ^2^	P	R	P	R	P	R
1:9	80.8	81.1	19.3	21.0	1.30	1.30	0.09	0.08
2:8	80.9	81.3	21.8	21.2	1.35	1.29	0.12	0.14
3:7	80.3	81.8	20.5	21.2	1.31	1.28	0.10	0.14
4:6	80.8	82.3	22.7	20.5	1.34	1.30	0.16	0.13
5:5	79.6	79.0	21.8	18.4	1.40	1.30	0.15	0.13

^1^ Perpendicular metallographic section to the main axis of the bar. ^2^ Parallel metallographic section to the main axis of the bar.

**Table 5:** The results of the chemical analysis.

Co:Ni Ratio	The State	Area of Analysis	Element Concentration					
			W		Ni		Co	
			wt. (%)	at. (%)	wt. (%)	at. (%)	wt. (%)	at. (%)
1:9	As sintered	matrix	41.35	18.38	52.50	73.09	6.16	8.54
2:8			41.79	18.66	46.68	65.28	11.54	16.06
3:7			43.03	19.45	40.20	56.90	16.78	23.66
4:6			43.47	19.74	34.02	48.37	22.53	31.9
5:5			43.95	20.06	29.75	42.53	26.30	37.43
		W-rich phase	73.46	46.98	10.65	21.33	15.90	31.70
1:9	Annealed 3 h at 1200 °C and water cooled	matrix	40.15	17.65	53.91	74.21	5.95	8.15
2:8			40.98	18.16	47.37	65.73	11.66	16.11
3:7			41.26	18.34	41.13	57.25	17.61	24.41
4:6			40.93	18.14	35.49	49.26	23.58	32.60
5:5			40.09	17.63	33.85	46.62	26.08	35.76
		W-rich phase	73.71	47.29	10.43	20.95	15.87	31.77

**Table 6:** The results of the calculated lattice parameter of the Ni base matrix.

Alloy Designation	Lattice Constant (nm)	
	As Sintered	Heat Treated 3 h at 1200 °C and Water Cooled
WNi9Co1	0.3598	0.3600
WNi8Co2	0.3603	0.3599
WNi7Co3	0.3609	0.3602
WNi6Co4	0.3607	0.3609
WNi5Co5	0.3612	0.3613

**Table 7:** The results of mechanical testing.

Co:Ni Ratio	Mechanical Properties			Hardenss	Young’s Modulus
	R_m_ (MPa)	R_p 0.2_ (MPa)	A_5_ (%)	HRC	GPa
After sintering					
1:9	626 ± 18	0	0	30.0	276 ± 13
2:8	650 ± 29	0	0	30.0	285 ± 11
3:7	663 ± 9	0	0	30.0	282 ± 9
4:6	773 ± 11.5	0	0	31.0	275 ± 14
5:5	262 ± 10	0	0	34.0	265 ± 15
Heat treated 3 h at a temperature of 1200 °C and water cooled					
1:9	998 ± 14	676 ± 26	25.3 ± 0.6	31.0	278 ± 12
2:8	1020 ± 23	725 ± 29	25.0 ± 1.5	30.5	286 ± 14
3:7	1022 ± 35	668 ± 42	27.2 ± 0.8	30.5	292 ± 16
4:6	852 ± 4.5	697 ± 32	6.5 ± 1	30.0	285 ± 12
5:5	343 ± 20	0	0	37.0	274 ± 7

**Table 8:** Results of microhardness measurements.

Co:Ni Ratio	Hardness			
	As Sintered		Heat Treated 3 h at a Temperature of 1200 °C	
	Tungsten Grain	Matrix	Tungsten Grain	Matrix
1:9	396 ± 35	370 ± 42	390 ± 37	402 ± 11
2:8	400 ± 15	367 ± 34	400 ± 25	358 ± 16
3:7	415 ± 23	410 ± 26	410 ± 15	390 ± 25
4:6	407 ± 19	377 ± 26	419 ± 17	383 ± 34
5:5	452 ± 24	490 ± 18	483 ± 26	497 ± 36
W-rich phase		936 ± 90		1030 ± 130

## Data Availability

Not applicable.

## References

[1] Magier M., Nowak A., Merda T., Żochowski P. (2017). Analysis Of Ballistic Characteristics Of 16th Century Arquebuses Used In Battle Of Pavia. Probl. Tech. Uzbroj..

[2] German R.M. Critical Developments in Tungsten Heavy Alloys. Proceedings of the 1st International Conference on Tungsten Tungsten Alloys.

[3] Lanz W., Odermatt W., Weihrauch G. Kinetic energy projectiles: Development history, state of the art, trends. Proceedings of the 19th International Symposium of Ballistics.

[4] Magness L.S., Kapoor D. Flow-softening tungsten composites for kinetic energy penetrator applications. Proceedings of the 2nd International Conference on Tungsten and Refractory Metals.

[5] Kruszka L., Janiszewski J., Grązka M. (2012). Experimental and Numerical Analysis of Al6063 Duralumin Using Taylor Impact Test.

[6] Hauver G.E., Melani A. (1990). Behavior of Segmented Rods during Penetration.

[7] Kennedy C., Murr L.E. (2002). Comparison of tungsten heavy-alloy rod penetration into ductile and hard metal targets: Microstructural analysis and computer simulations. Mater. Sci. Eng. A.

[8] Kumbhar K., Senthil P.P., Gogia A.K. (2017). Microstructural observations on the terminal penetration of long rod projectile. Def. Technol..

[9] Yadav S., Ramesh K.T. The dynamic behavior of a tungsten-hafnium composite for kinetic energy penetrator applications. Proceedings of the 4th International Conference on Tungsten Refractory Metals and Alloys.

[10] Magier M. (2018). The role of adiabatic shear bands effect in penetration process. Probl. Tech. Uzbroj..

[11] Kaczorowski M., Nowak W., Skoczylas P., Rafalski M. (2007). Zjawiska występujące w rdzeniach pocisków podczas penetracji płyty, Materiały konferencyjne, wydanie specjalne XVI konferencji naukowo-technicznej. Uzbrojenie.

[12] Arnold W. (2004). Tungsten heavy alloys for multiple impact applications. AIP Conf. Proc..

[13] Lassner E., Schubert W.D. (1999). Tungsten: Properties, Chemistry, Technology of the Element, Alloys, and Chemical Compounds.

[14] Majewski T. (2013). Technologiczne Uwarunkowania Właściwości Użytkowych Spieków Ciężkich W-(Fe, Ni, Re).

[15] Motyl K., Magier M., Borkowski J., Zygmunt B. (2017). Theoretical and experimental research of anti-Tank kinetic penetrator ballistics. Bull. Pol. Acad. Sci. Tech. Sci..

[16] Bless S.J., Tarcza K., Chau R., Taleff E., Persad C. (2006). Dynamic fracture of tungsten heavy alloys. Int. J. Impact Eng..

[17] Esquivel E.V., Murr L.E., Trillo E.A. (2003). Comparison of flow and shear band structures in oriented, columnar tungsten, single crystal tungsten-tantalum and sintered tungsten heavy alloy ballistic penetrators. Powder Metall..

[18] Das J., Appa Rao G., Pabi S.K. (2011). Deformation behaviour of a newer tungsten heavy alloy. Mater. Sci. Eng. A.

[19] German R.M. (1985). Liquid Phase Sintering.

[20] German R.M. (2022). Sintered tungsten heavy alloys: Review of microstructure, strength, densification and distortion. Int. J. Refract. Met. Hard Mater..

[21] Edmonds D.V. (1991). Structure-property relationships in sintered heavy alloys. Refract. Met. Hard Mater..

[22] Rabin B.H., Bose A., German R.M. (1989). Characteristics of Liquid Phase Sintered Tungsten Heavy Alloys. Int. J. Powder Met..

[23] Bose A., German R.M. (1988). Sintering atmosphere effects on tensile properties of heavy alloys. Met. Trans. A Phys. Met. Mater. Sci..

[24] Kunčická L., Kocich R., Klečková Z. (2020). Effects of Sintering Conditions on Structures and Properties of Sintered Tungsten Heavy Alloy. Materials.

[25] Panchal A., Venugopal Reddy K., Azeem P.A., Sarkar R., Paradkar A., Nandy T.K., Singh A.K. (2021). Effect of Ni/Fe ratio on microstructure, tensile flow and work hardening behaviour of tungsten heavy alloys in heat treated and swaged conditions. Philos. Mag..

[26] Satyanarayana P.V., Blessto B., Sokkalingam R., Rambabu C., Sivaprasad K. (2020). Effect of Fe Addition to Binder Phase on Mechanical Properties of Tungsten Heavy Alloy. Trans. Indian Inst. Met..

[27] Skoczylas P., Goroch O., Gulbinowicz Z., Barcz K., Kaczorowski M. (2019). Research into the Production of Tungsten Heavy Alloys with Specific Mechanical Properties, Problems of Mechatronics. Armament Aviat. Saf. Eng..

[28] Skoczylas P., Kaczorowski M. (2016). The influence of cyclic sintering on the structure and mechanical properties of Tungsten Heavy Alloy. Arch. Foundry Eng..

[29] Kaczorowski M., Skoczylas P., Krzyńska A., Kaniewski J. (2012). The strengthening of weight heavy alloys during heat treatment. Arch. Foundry Eng..

[30] Das J., Ravi Kiran U., Chakraborty A., Eswara N. (2009). Hardness and tensile properties of tungsten based heavy alloys prepared by liquid phase sintering technique. Int. J. Refract. Met. Hard Mater..

[31] Skoczylas P., Gulbinowicz Z., Goroch O. (2020). Microstructure and Properties of Tungsten Heavy Alloy Connections Formed during Sintering with the Participation of the Liquid Phase. Materials.

[32] Kaczorowski M., Skoczylas P. (2015). Kształtowanie struktury i właściwości wolframowych stopów ciężkich o przeznaczeniu specjalnym, Problemy Mechatroniki. Uzbroj. Lot. Inżynieria Bezpieczeństwa.

[33] Ravi Kiran U., Panchal A., Prem Kumar M., Sankaranarayana M., Nageswara Rao G.V.S., Nandy T.K. (2017). Refractory metal alloying: A new method for improving mechanical properties of tungsten heavy alloys. J. Alloys Compd..

[34] Yu Y., Erde W. (2004). Microstructure and properties of liquid phase sintered tungsten heavy alloys by using ultra-fine tungsten powders. Trans. Nonferrous Met. Soc. China.

[35] Natarajan S., Gopalan V., Rajan R.A.A., Jen C.-P. (2021). Effect of Rare Earth Metals (Y, La) and Refractory Metals (Mo, Ta, Re) to Improve the Mechanical Properties ofW–Ni–Fe Alloy—A Review. Materials.

[36] German R.M., Bose A., Kemp P.B., Zhang H., Basbarre T.G., Jandeska W.F. (1989). Additive Effect on the Microstructure and Properties of Tungsten Heavy Alloy Composites. Advances in Powder Metallurgy.

[37] Liu W., Ma Y., Huang B. (2008). Influence of minor elements additions on microstructure and properties of 93W–4.9Ni–2.1Fe alloys. Bull. Mater. Sci..

[38] Bose A., Sadangi R., German R.M. (2012). A review on alloying in tungsten heavy alloys. Suppl. Proc. Mater. Process. Interfaces.

[39] Tan X., Leng B., Qiu S. (2004). Influences of submicrometer Ta and Co dopants on microstructure and properties of tungsten heavy alloys. Trans. Nonferrous Met. Soc. China.

[40] Doepker S.P., Cuddy L.J. (1995). Aging studies and the associated changes in the microstructure of two W-Ni-Co alloys. Tungsten and Refractory Metals-1994, Proceedings of the International Conference on Tungsten and Refractory Metals.

[41] Prabhu G., Kumar R.A., Nandy T.K. (2021). Tungsten heavy alloys with two-phase matrix. Def. Sci. J..

[42] Penrice T.W., Bost J. (1988). High Density Tungsten-Nickel-Iron-Cobalt Alloys Heaving Improved Hardness and Method for Making Same. US Patent.

[43] Kiran U.R., Rao A.S., Sankaranarayana M., Nandy T.K. (2012). Swaging and heat treatment studies on sintered 90W-6Ni-2Fe-2Co tungsten heavy alloy. Int. J. Refract. Met. Hard Mater..

[44] Sunwoo A., Groves S., Goto D. (2006). Effect of matrix alloy and cold swaging on micro-tensile properties of tungsten heavy alloys. Mater. Lett..

[45] Skoczylas P., Goroch O., Gulbinowicz Z., Penkul A. (2021). The Effect of Cold Swaging of Tungsten Heavy Alloy with the Composition W91-6Ni-3Co on the Mechanical Properties. Materials.

[46] Hyung B.W., Hee H.M., Pyo K.E. (2006). Heat treatment behavior of tungsten heavy alloy. Solid State Phenom..

[47] Katavic B., Odanovic Z., Burzi M. (2008). Investigation of the rotary swaging and heat treatment on the behavior of W- and- phase in PM 92.5W-%-Ni-2.5Fe-0.26Co heavy alloy. Mater. Sci. Eng. A.

[48] Katavić B., Nikaćević M., Odanović Z. (2008). Effect of cold swaging and heat treatment on properties of the P/M 91W-6Ni-3Co heavy alloy. Sci. Sinter..

[49] Okamoto H., Massalski T.B. (1990). Binary Alloy Phase Diagrams.

[50] Murray J.L., Bennett L.H., Baker H., Massalski T.B. (1986). Binary Alloy Phase Diagrams.

[51] Senguptaa P., Mehera P., Panigrahia A., Mandala S., Basua S., Debataa M. (2021). Microstructure, distortion characteristics and mechanical behaviour of NiB modified 90W-6Ni-4Co heavy alloys. J. Alloys Compd..

